# Computer Intelligence in Modeling, Prediction, and Analysis of Complex Dynamical Systems

**DOI:** 10.1155/2015/948512

**Published:** 2015-08-05

**Authors:** Ivan Zelinka, Ajith Abraham, Otto Rossler, Mohammed Chadli, Rene Lozi

**Affiliations:** ^1^VŠB-Technical University of Ostrava, Ostrava, Czech Republic; ^2^University of Tubingen, Tubingen, Germany; ^3^University of Picardie Jules Verne, Amiens, France; ^4^Laboratoire J. A. Dieudonné, Université de Nice Sophia-Antipolis, Nice, France

Our technological civilization has had to confront numerous technological challenges such as finding the optimal solution of various problems including control technologies, power sources construction, and energy distribution amongst others. Analysis and prediction of complex system behavior such as stock exchange or other complex engineering structures and devices. Technology development of those and related areas has had and continues to have a profound impact on our civilization and lifestyle.

The topics discussed in this special issue belong to these mentioned areas and are mutually joined into a comprehensive text, which while discussing the specific selected topics gives a deeper insight to the interdisciplinary fusion of those modern and promising areas of emerging algorithms and technologies. This special issue discusses the mutual intersection of interesting fields of research, as artificial intelligence, unconventional algorithm, complex system behavior like chaos, soft computing, simulators, and software engineering amongst others. Novel techniques are also discussed in this special issue, which are able to handle tasks such as model and control of various systems, optimization by means standard and novel methods. Together with many interesting emerging technologies, a reader will also find in the special issue various mathematical and algorithmical methods used for proposed applications.

Therefore, this special issue is a timely volume to be welcome by the community focused on above mentioned techniques and beyond. This special issue is devoted to the studies of common and related subjects in intensive research fields of modern algorithms and their applications. For these reasons, we believe that this special issue will be interesting to scientists and engineers working in the above mentioned fields of research and applications.

*Ivan  Zelinka* *Ivan  Zelinka* *Ajith  Abraham* *Ajith  Abraham* *Otto  Rossler* *Otto  Rossler* *Mohammed  Chadli* *Mohammed  Chadli* *Rene  Lozi* *Rene  Lozi*

